# Sustainable Valorization of Palm Fatty Acid Distillate into Green Diesel Using Ni–Co Catalysts Supported on Zeolite from Kaolin Waste under Solvent- and Hydrogen-Free Conditions

**DOI:** 10.1021/acsomega.5c08463

**Published:** 2025-11-08

**Authors:** Brenda Fernanda Honorato de Oliveira, Bruno Marques Viegas, Mauricio Velasquez, Emanuel Negrão Macêdo

**Affiliations:** † Engineering of Natural Resources of the Amazon, 37871 Federal University of Pará, Belém, Pará 66075-110, Brazil; ‡ Graduate Program in Biotechnology, Federal University of Pará, Belém, Pará 66075-110, Brazil; § Estado Sólido Y Catálisis Ambiental (ESCA), Departamento de Química, Facultad de Ciencias, Universidad Nacional de Colombia, Carrera 30 No. 45-03, Bogotá 057, Colombia

## Abstract

The use of low-cost, nonedible feedstocks has proven to be a promising route for catalytic deoxygenation reactions aimed at producing diesel-like hydrocarbons. In this study, palm fatty acid distillate, a byproduct of crude palm oil refining, was converted into green diesel via thermal and catalytic deoxygenation using zeolite-supported Ni, Co, and NiCo catalysts (10 wt % metal, synthesized from kaolin residue). The reactions were carried out at 250–350 °C for 30 min without solvent or hydrogen. The process was evaluated using thermogravimetric analysis, and kinetic parameters were estimated via the Coats–Redfern method. Both the thermal and catalytic routes yielded 60–65% organic liquid products, with 77–83% hydrocarbons in the C_8_–C_17_ range. The catalytic route resulted in high yields of diesel-range hydrocarbons (C_15_–C_17_), with 86% for NiZ, 75% for CoZ, and 65% for NiCoZ, whereas the thermal route reached only 45%. Thermodynamic evaluation further demonstrated that cobalt-based catalysts enhanced deoxygenation selectivity while limiting secondary cracking. This study presents a sustainable and scalable approach. This study presents a sustainable and scalable approach for converting industrial waste into high-quality green diesel using waste-derived catalysts under mild, hydrogen-free conditions.

## Graphical abstract

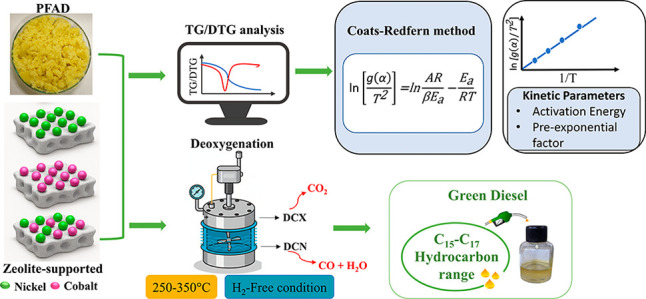

## 1 Introduction

The increasing global energy demand and the environmental impact of fossil fuel combustion have driven the search for alternative and sustainable energy sources. In this context, biofuels derived from renewable biomass have emerged as a viable strategy to reduce greenhouse gas emissions and decrease reliance on petroleum-based fuels.^1,2^ Recent studies have highlighted the feasibility of employing nonedible oils as feedstocks for biofuel production.^3^ Among these, agro-industrial residues have attracted particular interest, as they do not compete with the food supply chain and exhibit significant potential for conversion into renewable liquid fuels.^4,5^

Palm fatty acid distillate (PFAD), a low-value byproduct from the physical refining of crude palm oil, stands out as a promising feedstock for green diesel production.^6^ PFAD is generated as approximately 5% of crude palm oil (CPO) during its refining process.^7^ In 2024, CPO production in Brazil reached 600,000 tons,^8^ which corresponds to an estimated 30,000 tons of PFAD, based on production volumes and typical acidity levels, reinforcing the availability of this feedstock for scalable applications.

Among the technological routes for converting FFAs into hydrocarbons, catalytic cracking, hydrodeoxygenation (HDO), and deoxygenation (DO) have shown promising results. HDO refers to the removal of oxygen through hydrogenation reactions, requiring an external hydrogen supply, while DO encompasses hydrogen-free pathways such as decarboxylation and decarbonylation, where oxygen is released mainly as CO, CO_2_, and H_2_O.^9,10^ Although HDO is often regarded as the most efficient route for obtaining high-quality hydrocarbons, its dependence on hydrogen increases both cost and environmental impact. In contrast, DO has gained particular interest due to its lower operational costs, reduced environmental footprint, and potential scalability for green diesel production.^11,12^ The resulting fuels typically exhibit high cetane numbers as well as favorable thermal and oxidative stability, making them compatible with, or even superior to, conventional fossil diesel.^13^ Beyond adding value to this agro-industrial residue, this route constitutes a promising pathway for the partial substitution of petroleum-derived diesel, with the potential to achieve a 40–80% reduction in CO_2_ emissions relative to fossil diesel.^14^

Regarding catalytic systems, controlled reduction of the catalyst is essential to optimize properties such as acidity and, consequently, to enhance both the efficiency and selectivity of the deoxygenation process for green diesel production.^15^ In this context, non-noble metals such as nickel (Ni) and cobalt (Co), in their reduced forms, have been extensively investigated due to their low cost, abundance, acid–base properties, and catalytic activity.^16–18^ Furthermore, bimetallic systems such as NiCo have attracted considerable attention for their synergistic effects that improve catalytic performance, although challenges related to sintering and carbon deposition remain.^19^ Acidic materials such as zeolites improve metal dispersion and promote desirable reactions such as isomerization, in addition to providing high thermal stability.^20^ Zeolites can be synthesized using various waste materials,^21,22^ the synthesis of zeolites from industrial residues like kaolin represents an added sustainability benefit, aligning with circular economy principles.

From a kinetic standpoint, thermogravimetric analysis (TGA) is widely used to evaluate the thermal behavior of organic materials and to estimate parameters such as activation energy and pre-exponential factor, which are essential for understanding thermochemical pathways.^23,24^ The Coats–Redfern method has proven to be reliable for determining these parameters in thermal and catalytic processes.^25^

In this context, the present work aims to investigate green diesel production from PFAD, employing reduced Ni, Co, and NiCo catalysts supported on zeolite derived from kaolin waste. The innovation lies in operating under solvent- and hydrogen-free conditions, demonstrating that catalytic reduction optimizes deoxygenation selectivity. Kinetic analysis via thermogravimetry elucidates the reaction mechanism, paving the way for more economical, scalable, and sustainable biofuel routes.

## 2 Materials and Methods

### 2.1 Materials

The palm fatty acid distillate used in this work was supplied by Agropalma S/A Company (Belém, Brazil). For zeolite synthesis, natural kaolin waste from the tailings pond of an industrial facility in the Amazon region was obtained. To prepare the metallic catalysts, nickel nitrate (Ni­(NO_3_)_2_·3H_2_O, 97%, Vetec, São Paulo, Brazil) and cobalt nitrate (Co­(NO_3_)_2_·H_2_O, 97%, Dinâmica, São Paulo, Brazil) were selected as the metal precursors.

### 2.2 Catalyst Preparation

Zeolite was synthesized from a waste material obtained from kaolin production, according to the methodology reported in previous works,^26,27^ with additional details on the synthesis and physicochemical characterization provided in a prior publication.^5^

Metallic catalysts (Ni, Co, and NiCo) were prepared with a nominal metal loading of 10 wt % on zeolite using the wet impregnation method. Aqueous solutions of the corresponding metal nitrates were mixed with zeolite and stirred at room temperature for 4 h, followed by heating at 70 °C for 8 h and drying at 90 °C. The resulting solids were calcined at 500 °C for 2 h in air (flow rate: 50 mL min^–1^), macerated, and sieved through a 100-mesh ASTM screen. Subsequently, the catalysts were reduced at 550 °C for 2 h under a hydrogen atmosphere (50 mL min^–1^) with a heating rate of 10 °C min^–1^. The reduced materials were designated as NiZ, CoZ, and NiCoZ.

### 2.3 Catalyst Characterization

The structural properties of catalysts were characterized by X-ray diffraction (XRD) using a Bruker diffractometer (model D8 with Bragg–Brentano, Billerica, MA, USA) with a Cu anode (Kα1 = 1.540598 Å) operated at 40 kV and 40 mA. The diffraction patterns were recorded in the 2θ range of 5–75° with a step interval of 0.02° and a period of 0.2 s. The crystalline phases in the samples were identified and compared to powder diffraction files (PDF): PDF 901-2985 (nickel), PDF 015-0806 (cobalt). Data were acquired using the software X’Pert Data Collector and treated using the software X’Pert HighScore Plus (PANalytical), based on data from the International Centre for Diffraction Data (ICDD).

Scanning electron microscopy (SEM) was used to analyze the morphological characteristics of the synthesized catalysts. The analyses were performed using a high-resolution VEGA microscope (Shimadzu), following standard preparation procedures. Before taking images, the samples were deposited onto a graphite tape and coated with a nanofilm of gold.

Temperature-programmed reduction with hydrogen (H_2_-TPR) was carried out using a CHEMBET 3000 QUANTACHROME analyzer equipped with a thermal conductivity detector. In this analysis, hydrogen was used as reducing gas and argon as the purge and carrier gas. The heating rate was 10 °C min^–1^. The samples were previously degassed at 450 °C for 1 h. Hydrogen consumption was calculated from a calibration curve using CuO as the standard.

### 2.4 Thermogravimetric Analysis

Thermogravimetric analysis was performed using a Hitachi NEXTA STA300 instrument, with the operating temperature ranging from 25 to 700 °C at a constant heating rate of 15 °C min^–1^ under a nitrogen atmosphere (flow rate: 100 mL min^–1^). Samples of approximately 10 mg were placed in a platinum crucible, and catalysts were added at a concentration of 5 wt % relative to the PFAD mass. The experiments were conducted both with and without catalysts to evaluate their influence on the thermal decomposition behavior of the feedstock.

### 2.5 Calculation of Kinetic Parameters

The decomposition process of PFAD is a heterogeneous chemical reaction, and the kinetic parameters can be determined from TGA data. The mass loss of samples can be expressed by eq 1:^28^

1 $$\alpha =\frac{m_{0}-m\left(t\right)}{m_{0}-m_{f}}$$

where α is the conversion rate, *m*_0_ is the initial mass of the sample, *m* is the mass of the sample at any decomposition time *t*, and the final mass *m*_f_ after decomposition. TGA of PFAD was performed under nonisothermal conditions, characteristic of a pyrolysis process. Following mathematical derivation, the reaction kinetics can be evaluated using the Arrhenius approach, and the reaction rate is expressed by eq 2:

2 $$\frac{d\alpha }{dt}=k\left(T\right)f\left(\alpha \right)=Ae^{\left(-\frac{E_{a}}{RT}\right)}f\left(\alpha \right)$$

where *A* denotes the pre-exponential factor (min^–1^), *E*_a_ denotes the apparent activation energy (kJ mol^–1^), *R* is the universal gas constant (8.314 J mol^–1^ K^–1^), *T* is the absolute temperature (K), and *f*(α) is the mechanism function that characterizes the reaction model based on the rate-controlling step. Considering that the heating rate d*T*/d*t* can be defined as β, the rate expression can be reformulated as shown in eq 3.

3 $$\beta \frac{d\alpha }{dt}=Ae^{\left(-\frac{E_{a}}{RT}\right)}f\left(\alpha \right)$$

If the integral form of the reaction model $g\left(\alpha \right)=\int_{0}^{\alpha }d\alpha /f\left(\alpha \right)$ , eq 3 can be rewritten in its integrated form as presented in eq 4:

4 $$g\left(\alpha \right)=\int_{0}^{\alpha }\frac{d\alpha }{f\left(\alpha \right)}=\frac{A}{\beta }\int_{T_{0}}^{T}e^{\left(-\frac{E_{a}}{RT}\right)}dT$$

where *g*(α) is the integral form of *1*/*f*(α). To determine the kinetic parameters of thermal decomposition, two primary mathematical approaches are commonly employed: the model-free (isoconversional) method and the model-fitting (model-based) method.^29^ The latter allows for simultaneous estimation of both the apparent activation energy (*E*_a_) and the pre-exponential factor (*A*) from a single heating rate. Among the model-fitting approaches, the Coats–Redfern method is widely adopted due to its simplicity and low relative error compared to alternative techniques. The mathematical formulation of this method is given by eq 5.^30,31^

5 $$\ln\left[\frac{g\left(\alpha \right)}{T^{2}}\right]=\ln\frac{AR}{\beta E_{a}}-\frac{E_{a}}{RT}$$

According to eq 5, a linear relationship can be established by plotting ln­[*g*(α)/*T* ^2^] vs 1000/*T*, where the slope corresponds to −*E*_a_/*R* and the intercept to ln­[*AR*/β*E*_a_]. An accurate selection of the reaction mechanism function *f*(α) is critical for the reliable estimation of kinetic parameters in pyrolysis processes. Qiao et al.^25^ compiled the most adopted kinetic models for such analyses. In this study, the reaction order model F1.5 was applied, the best-fitting model for oils according to the literature,^25^ as defined by eqs 6and 7.

6 $$f\left(\alpha \right)={\left(1-\alpha \right)}^{3/2}$$

7 $$g\left(\alpha \right)=2\left[{\left(1-\alpha \right)}^{-1/2}-1\right]$$

These equations enable the evaluation of reaction kinetics and the determination of activation energy without requiring prior knowledge of the exact reaction order, thereby simplifying the modeling of the thermal decomposition process.

### 2.6 Reaction Conditions

A hydrogen-free deoxygenation reaction was conducted in a batch reactor to convert PFAD into green diesel, as illustrated in Figure 1.

**Figure 1:**
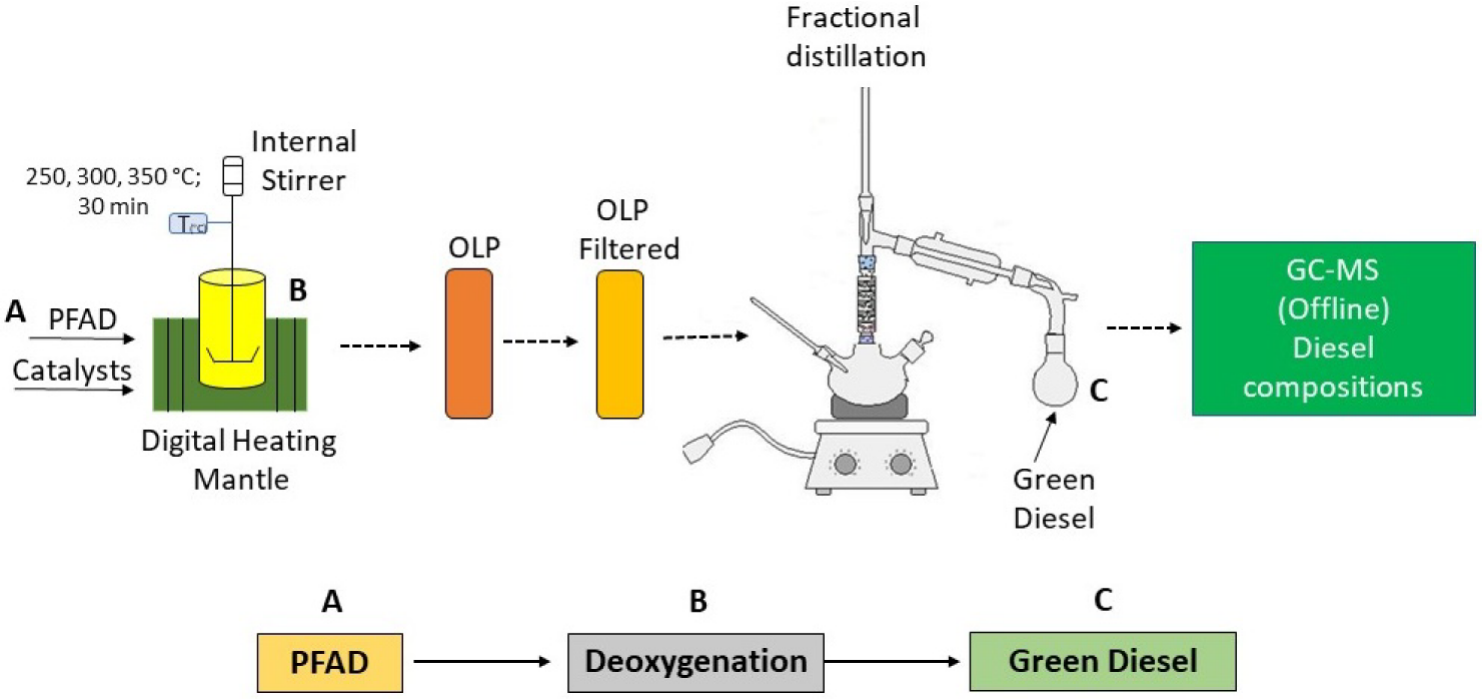
Process flow diagram of the deoxygenation reaction of PFAD in a batch system.

Reactions were carried out in a 100 mL Parr batch reactor (model 4590, Parr Instrument Company, Moline, IL, USA) equipped with mechanical stirring and temperature control. The reactor was charged with 40 g of PFAD and a catalyst loading of 5 wt % relative to the substrate. The reaction pressures were autogenous, resulting solely from the vapor pressure of the reactants at the reaction temperature. Experiments were conducted at 250, 300, and 350 °C, with a heating rate of 5 °C min^–1^, stirring at 600 rpm, and a reaction time of 30 min. After completion, the mixture was filtered, and the yields of organic liquid products (OLPs) and solid residues were determined using eq 8.

8 $$Y\;\left(\%\right)=\frac{m_{fraction}}{m_{initial}}\times 100$$

where *m*_fraction_ corresponds to the mass of the liquid organic products or solid residues, and *m*_initial_ is the initial mass of PFAD. The yield of gaseous products was determined by mass balance, as the difference between the initial mass and the sum of the liquid and solid fractions. The OLPs were subjected to fractional distillation, and the resulting products (distillates between 200 and 300 °C) were identified and quantified by gas chromatography. After the reaction, the catalyst was washed with hexane and recovered for further analysis.

### 2.7 Identification and Quantification of PFAD and Reaction Products

Following the distillation of PFAD, the reaction products were quantified by gas chromatography using a Thermo Trace GC-Ultra chromatograph equipped with a flame ionization detector (FID) and a CP-Sil 5 CB column (30 m × 0.53 mm × 1.5 μm). Prior to quantification, the samples were esterified and compared with methyl ester standards using a gas chromatograph coupled to a mass spectrometer (Shimadzu GC-MS-2010 with QP2010 interface and electron impact ionization, Kyoto, Japan), equipped with a DB-5 capillary column (30 m × 0.25 mm × 0.25 μm). The hydrocarbon yield and selectivity resulting from deoxygenation reactions are discussed in detail in a previous study.^5^

## 3 Results and Discussion

### 3.1 Catalyst Characterization

The XRD patterns of the zeolite support and the reduced catalysts are shown in Figure 2.

**Figure 2:**
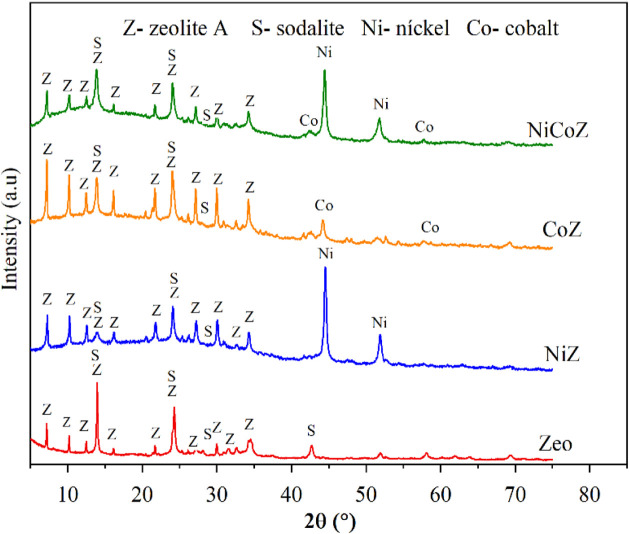
X-ray diffraction patterns of the zeolite support (Zeo) and the reduced catalysts NiZ, CoZ, and NiCoZ. The main diffraction peaks are assigned to zeolite A (Z), sodalite (S), nickel (Ni), and cobalt (Co).

The support pattern shows the characteristic peaks of zeolite A and sodalite. Zeolite A belongs to the class of aluminosilicate molecular sieves composed of sodalite cage units connected by four-membered rings, forming a three-dimensional porous framework. The detection of sodalite peaks suggests that the synthesis of zeolite A was only partially selective. In the diffractograms of the reduced catalysts, reflections corresponding to metallic Ni and Co phases are clearly observed, confirming the effectiveness of the reduction process. For the nickel catalyst (NiZ), diffraction peaks at 44.56° and 51.97° were assigned to metallic nickel with a cubic structure, while the cobalt catalyst (CoZ) displayed peaks at 42.72° and 52.69°, consistent with the cubic crystalline structure of metallic cobalt.^32,33^ The bimetallic catalyst (NiCoZ) presented diffraction signals for both Ni^0^ and Co^0^ phases. Notably, the relative intensity of the Co^0^ reflections was lower than that of Ni^0^, which may be attributed to the higher mass attenuation coefficient of cobalt, leading to greater absorption of X-ray photons compared to nickel.

The average crystallite sizes of the nickel, cobalt, and bimetallic nickel–cobalt catalysts were estimated using the most intense diffraction peaks, located at 2θ = 44.50° for nickel and 2θ = 42.28° for cobalt. The calculations were performed using the Scherrer equation, and the results are summarized in Table 1.

**Table 1:** Average Crystallite Sizes of the Zeolite Support and Catalysts

Catalyst	Ni crystallite size (nm)	Co crystallite size (nm)
ZeO	-	-
NiZ	32.29	-
CoZ	-	10.46
NiCoZ	36.34	14.65

The crystallite size of the NiZ is larger than the particle size of the CoZ, which may be attributed to a lower dispersion of the Ni^0^ particles on the zeolite support. The average crystallite size observed for NiZ exceeded the values reported by Peng et al.,^33^ which ranged from 8 to 18 nm. According to Romero et al.,^34^ crystallite size can vary depending on the nickel loading and the strength of the metal–support interaction. This behavior was further confirmed by Shi et al.,^35^ who demonstrated that varying nickel concentrations in HZSM-5 affected dispersion and led to differences in crystallite size under distinct conditions. Regarding CoZ, the crystallite size was consistent with the values reported by Horváth et al.^36^ (12–26 nm), which suggests that the reduction temperature plays a significant role in determining the final particle size. The bimetallic catalyst NiCoZ exhibited Ni and Co crystallite sizes like those of the corresponding monometallic systems.

As reported in previous studies, the morphology of the zeolite support exhibits polycrystalline sodalite structures resembling woolen balls and cubic crystals characteristic of zeolite A.^36^ Figure 3 presents SEM-EDS micrographs of the support (Figure 3a) and the corresponding catalysts after metal impregnation and reduction. Overall, it is possible to observe the formation of fine particles distributed on the surfaces of sodalite and zeolite A structures. Nevertheless, distinct morphological features can be noted among the catalysts. NiZ (Figure 3b) exhibits spherical aggregates, whereas CoZ (Figure 3c) presents the formation of small sheet-like structures. In the case of the bimetallic catalyst NiCoZ (Figure 3d), the morphology is also predominantly composed of small sheets, indicating that cobalt may exert greater influence on the surface structure under the synthesis conditions applied.

**Figure 3:**
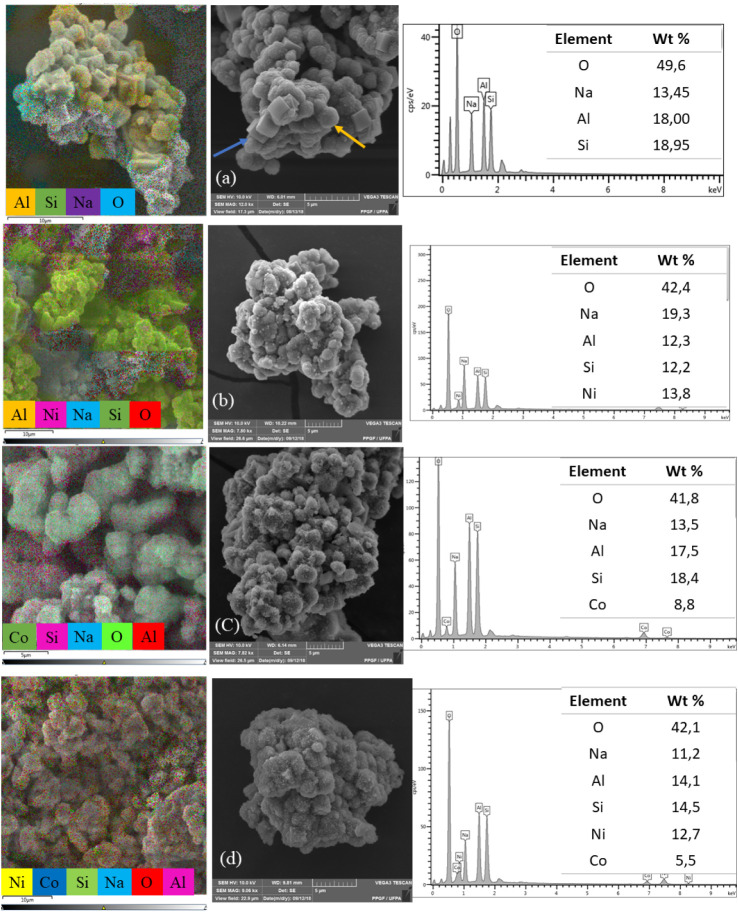
SEM images and EDS elemental mapping of the catalysts (a) Zeo, (b) NiZ, (c) CoZ, and (d) NiCoZ.

The TPR-H_2_ profiles of the catalysts are presented in Figure 4. The NiZ catalyst displays a broad reduction peak comprising three distinct contributions, suggesting the presence of multiple nickel oxide (NiO) species with varying degrees of interaction with the support.

**Figure 4:**
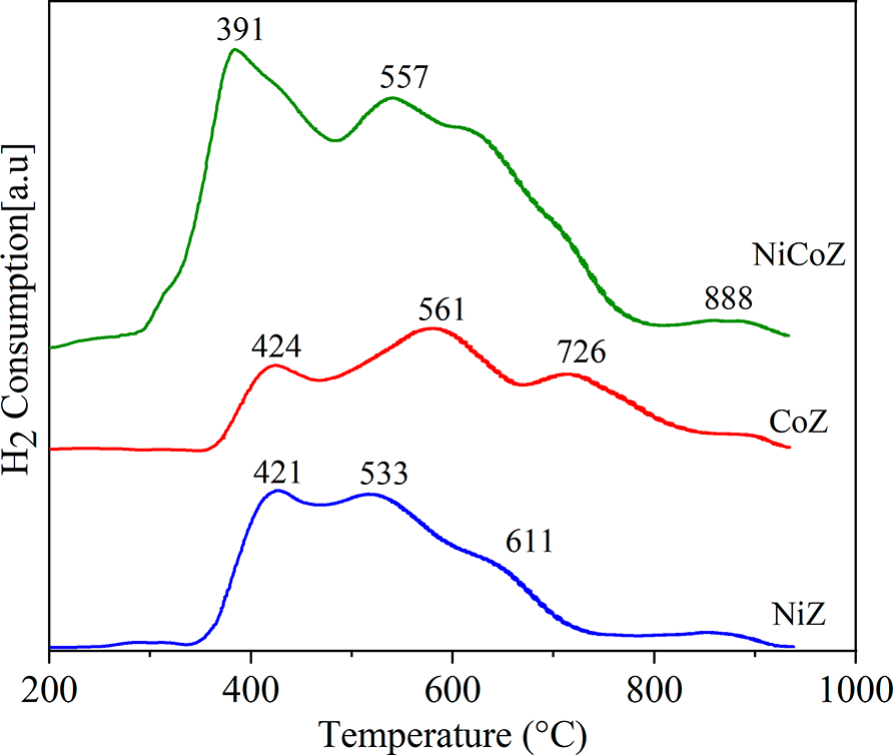
H_2_-TPR profiles of the catalysts.

The reduction of NiO under a hydrogen atmosphere proceeds via two sequential steps, as described in eqs 9 and 10.^37^

9 $$2\mathrm{Ni}O+H_{2}\to {\mathrm{Ni}}_{2}O+H_{2}O$$

10 $${\mathrm{Ni}}_{2}O+H_{2}\to 2{\mathrm{Ni}}^{0}+H_{2}O$$

The interaction strength between Ni^2+^ and the sodalite or zeolite A structures may vary depending on whether the cations are located within or outside the zeolite framework, which influences their reducibility.^38^ Accordingly, the first reduction peak observed at 421 °C is attributed to NiO species in more accessible regions of the catalyst, with weaker interactions with the support material.^39^ The second peak, appearing at 530 °C, likely corresponds to smaller NiO particles that exhibit stronger interaction with the support, potentially due to the formation of nickel aluminates (NiAl_2_O_4_ or Ni_2_AlO_4_) during the calcination step.^40^ A third, less intense peak at 611 °C indicates the presence of even stronger metal–support interactions, attributed to the coordination between Ni and Al^3+^ species in the zeolite matrix.^41^ In the case of cobalt-based catalysts, reduction occurs through consecutive steps, involving the transformation of Co_3_O_4_ to CoO and subsequently from CoO to Co^0^.^41^ The reduction of Co_3_O_4_ can be represented as described in the following eqs 11–13.^42,43^

11 $$6\mathrm{Co}O\left(OH\right)+H_{2}\to 2{\mathrm{Co}}_{3}O_{4}+4H_{2}O$$

12 $${\mathrm{Co}}_{3}O_{4}+H_{2}\to 3\mathrm{Co}O+H_{2}O$$

13 $$\mathrm{Co}O+H_{2}\to {\mathrm{Co}}^{0}+H_{2}O$$

The reduction profile of the CoZ sample shows two main peaks. The first one, at approximately 425 °C, is associated with reducing the Co^3+^ to Co^2+^, reflecting the transformation of Co_3_O_4_ into CoO. The second peak, observed at 561 °C, is attributed to the subsequent reduction of CoO to metallic Co.^36^ Additionally, a third peak appears at a higher temperature (726 °C), which may be associated with cobalt species possessing smaller crystallite sizes and exhibiting stronger metal–support interactions.^41,44^ These results suggest that nickel oxides undergo reduction more readily than cobalt oxides, particularly during the initial reduction stage.

The bimetallic NiCoZ catalyst shows an important decrease in the reduction peaks. In this profile, the first peak was shifted to 391 °C and the second to 557 °C. Similar behavior has been reported in the literature,^38^ which demonstrated that the incorporation of cobalt facilitates the reduction of nickel oxides, likely due to the formation of spinel-type structures. Furthermore, the authors indicated that the nickel reducibility increased from 82% in the monometallic catalyst to 94% in the presence of an equimolar amount of cobalt. This synergistic effect enhances the catalytic performance of the bimetallic system by promoting more efficient reduction of the active metal species.^45^

### 3.2 PFAD Composition

The chemical composition of the PFAD used in this study is presented in Table 2. The gas chromatographic analysis of PFAD composition is shown in Figure S1 (see Supporting Information).

**Table 2:** Physicochemical Properties and Chemical Composition of PFAD

Properties	PFAD
Acid value (mg KOH g^–1^)	144.7
Viscosity at 40 C (cSt)	20.8
Calorific value (MJ kg^–1^)	41.5
**Fatty acid composition of oil (%)**	
Lauric acid (C12:0)	1.42
Myristic acid (C14:0)	1.40
Palmitic acid (C16:0)	50.12
Stearic acid (C18:0)	7.43
Oleic acid (C18:1)	35.03
linoleic acid (C18:2)	4.57

Approximately 97% of its composition is formed by free fatty acids, predominantly comprising C_16_ (palmitic acid, 50%) and C_18_ species (47%), such as stearic, oleic, and linoleic acids. These FFAs can be thermochemically converted into hydrocarbons through cracking and deoxygenation reactions. In decarboxylation pathways, C_16_ and C_18_ fatty acids yield *n*-pentadecane (C_15_H_32_) and *n*-heptadecane (C_17_H_36_), with CO_2_ as a byproduct due to oxygen removal. Alternatively, decarbonylation reactions lead to the formation of *n*-pentadecenes and *n*-heptadecenes, generating CO and H_2_O. Moreover, the presence of Brønsted acid sites on the catalyst support can promote cracking reactions, favoring the production of lighter hydrocarbons (C_8_–C_10_).^10,46^

### 3.3 Thermogravimetric Analysis

Figure 5 presents the TGA and corresponding DTG curves of PFAD and PFAD with catalysts during pyrolysis. These profiles illustrate the mass loss behavior and decomposition stages under thermal treatment.

**Figure 5:**
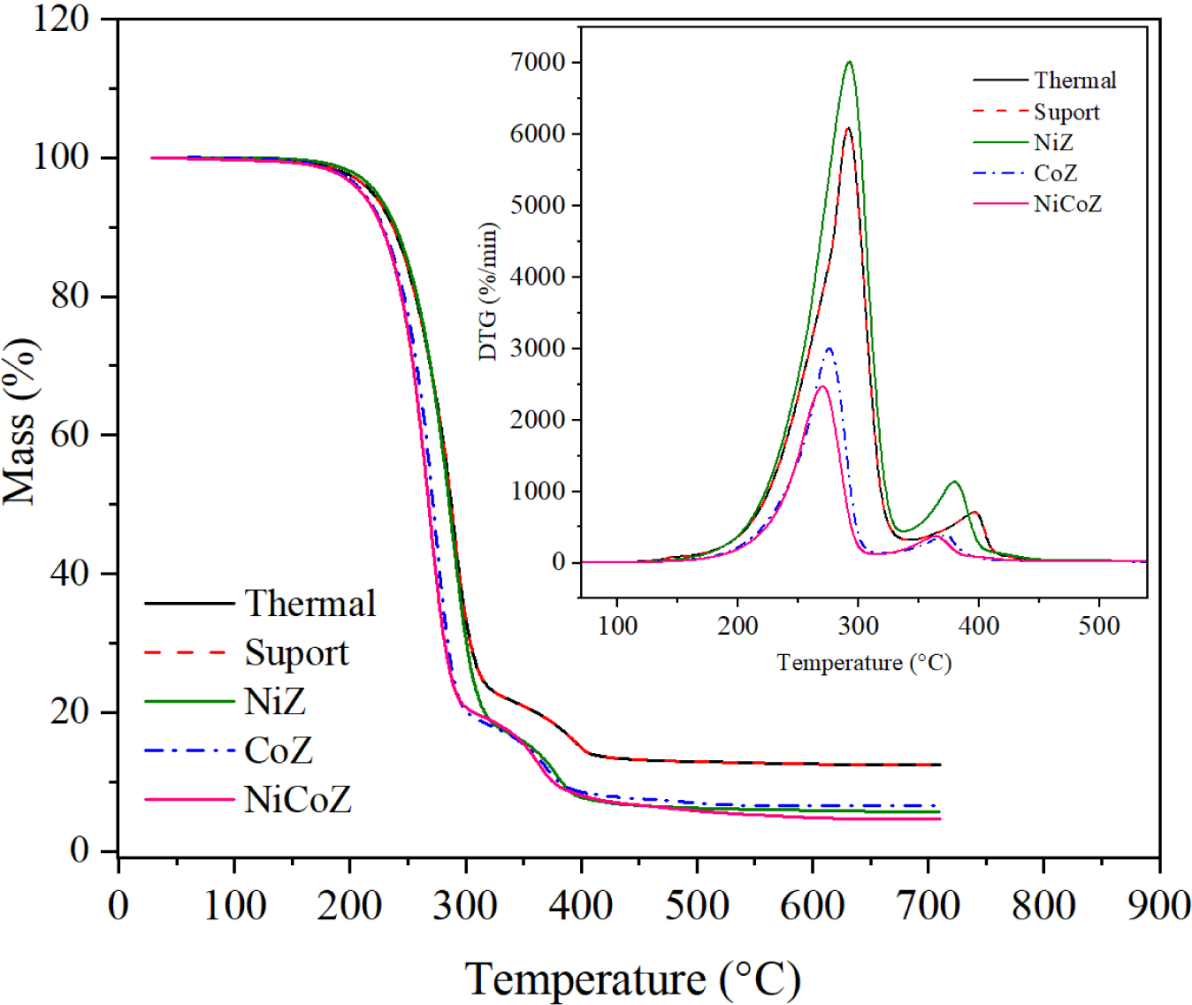
TGA and DTG curves of PFAD deoxygenation at a heating rate of 15 °C min^–1^.

Figure 5 shows that the TGA profiles of PFAD, with or without catalysts, exhibit similar thermal decomposition behavior, although the extent of mass loss differs among the samples. The DTG curves display narrow and sharp peaks, indicating a rapid mass loss within a restricted temperature range, predominantly between 200 and 320 °C. For the thermal process, support, and NiZ catalyst, the DTG peaks are centered around 290 °C, while for CoZ and NiCoZ, they appear near 270 °C, suggesting enhanced reactivity at lower temperatures. The maximum decomposition rates are observed at the temperatures corresponding to the DTG peak positions. The thermal degradation of PFAD occurs over the range of 200 to 405 °C, with the most pronounced mass loss occurring near 300 °C. The TGA curves confirm substantial thermal decomposition with a minimal residual fraction remaining after pyrolysis.

### 3.4 Kinetic Study

The range of α was set from 0.1 to 0.8, with increments of 0.10, for the calculation of kinetic parameters. Figure 6 shows the fitting plots obtained using the Coats–Redfern method at the selected α values.

**Figure 6:**
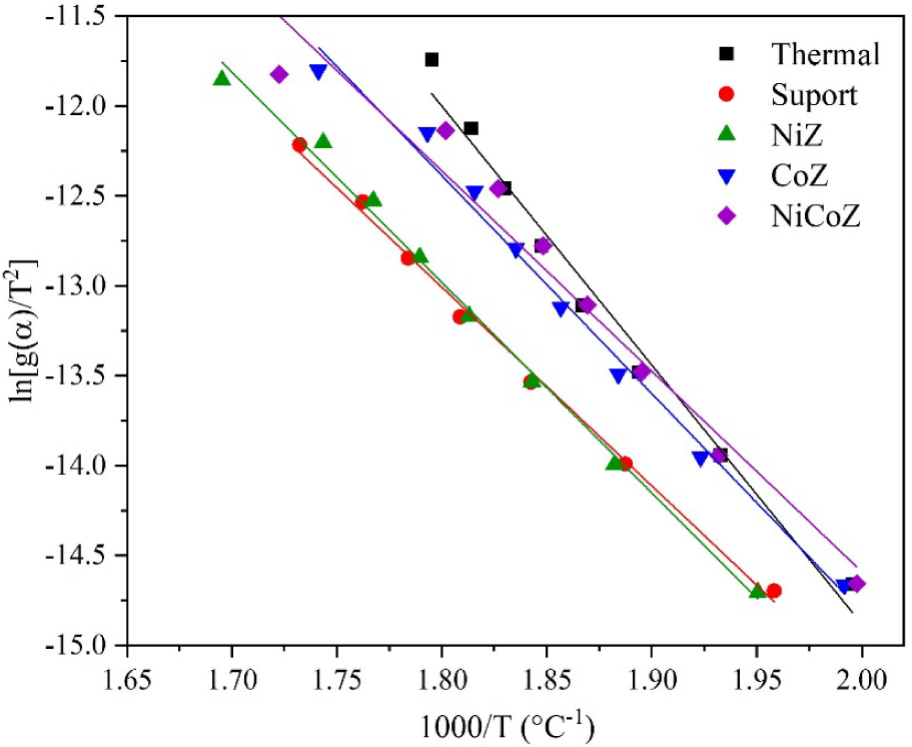
Fitting plot obtained using the Coats–Redfern method.

Based on the linear regressions in Figure 6, the kinetic parameters reported in Table 3 were determined. For PFAD deoxygenation, the apparent activation energy and the pre-exponential factor were calculated as 119.67 kJ mol^–1^ and 2.37 × 10^8^ s^–1^, respectively.

**Table 3:** Kinetic Parameters of PFAD Deoxygenation

Samples	*E*_a_ (kJ mol^–1^)	*A* (s^–1^)	Equation	*R* ^2^
PFAD	119.67	2.37 × 10^8^	*y* = −14.39*x* + 13.91	0.9824
Support	91.84	1.61 × 10^5^	*y* = −7.54*x* + 0.41	0.9961
NiZ	97.25	5.63 × 10^5^	*y* = −11.70*x* + 8.07	0.9941
CoZ	100.79	2.28 × 10^6^	*y* = −12.12*x* + 9.44	0.9892
NiCoZ	92.65	3.69 × 10^5^	*y* = −11.14*x* + 7.70	0.9613

The incorporation of catalysts into the PFAD deoxygenation system resulted in reduced activation energy and pre-exponential factor values when compared to the noncatalyzed reaction. The values of *E*_*a*_ and *A* were 91.84 kJ mol^–1^ and 1.61 × 10^5^ s^–1^ for the support, 97.25 kJ mol^–1^ and 5.63 × 10^5^ s^–1^ for NiZ, 100.79 kJ mol^–1^ and 2.28 × 10^6^ s^–1^ for CoZ, and 92.65 kJ mol^–1^ and 3.69 × 10^5^ s^–1^ for NiCoZ, respectively. Among these, the support and the bimetallic catalyst showed the lowest activation energy values. As reported in previous studies, pre-exponential factors lower than 10^9^ s^–1^ are generally associated with surface-controlled reaction mechanisms or the presence of complex molecular structures. In contrast, values exceeding 10^9^ s^–1^ typically indicate the prevalence of straight-chain compounds.^47^ The *A* values obtained for PFAD and for all catalytic systems remained below 10^9^ s^–1^, reinforcing that the pyrolysis reactions are predominantly governed by surface-controlled kinetics and reducing the pre-exponential factor with the use of catalysts.

### 3.5 Catalytic Activity

The yields of OLPs, gaseous products, and solid residues from the thermal and catalytic experiments conducted at different temperatures are shown in Figure 7.

**Figure 7:**
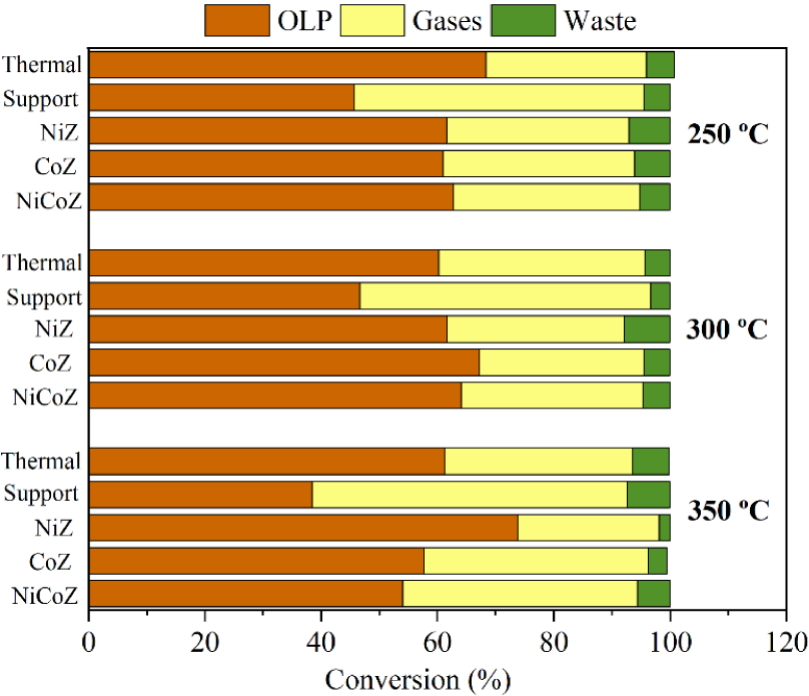
Yields of liquid, gaseous and solid products from deoxygenation reactions at different temperatures.

As previously reported by De Oliveira et al.,^5^ all thermal reactions performed in the absence of catalyst yielded between 60 and 68% of OLPs and approximately 27% of gaseous products, indicating a thermodynamically favorable conversion of PFAD. Similar values were observed for palm oil under thermal cracking conditions,^48^ confirming that noncatalytic reactions generally lead to comparable product distributions. However, based on the OLP distribution after fractional distillation, the resulting biofuel exhibits low quality due to the formation of cracked and oxygenated compounds.^16,49^ In contrast, the reaction with the zeolite support produced OLP yields below 45%, highlighting the influence of acidity on the formation of gaseous products. According to Matharasi et al.,^50^ the evaluation of different zeolites demonstrated gas yields ranging from 20 to 50%, indicating that pore size, morphology, and acidity are decisive parameters in the process. As also highlighted in the literature,^51^ both acidic and basic sites contribute to deoxygenation and cracking activity, although excessive acidity or basicity can intensify C–C bond cleavage and consequently reduce liquid product yields.

At all evaluated temperatures, the catalysts exhibited higher OLP yields compared to the zeolite support. This behavior suggests that metal incorporation into the support reduces the density of surface acid sites, thereby lowering the formation of gaseous products and promoting reactions such as deoxygenation and decarboxylation. Overall, the catalysts presented similar OLP yields at 250 and 300 °C, ranging from 60 to 65%. However, a marked increase in OLP yield was observed for NiZ at 350 °C, reaching 73.8%, while no significant improvements were noted for CoZ and NiCoZ under the same condition. The data also indicates substantial gas formation at this temperature (35%), although the specific composition was not identified in this study. These gaseous products are likely associated with H_2_, CO_*X*_, H_2_O, and low-molecular-weight hydrocarbons, and this limitation is acknowledged. As for the solid residues, yields were below 7% for all reactions and were primarily composed of unconverted free fatty acids.

### 3.6 Analysis of Products

Based on the composition of PFAD, the deoxygenation reaction is expected to yield a high proportion of diesel-range hydrocarbons, particularly C_15_ and C_17_ compounds.^10^ Therefore, a comparative analysis of hydrocarbon selectivity was carried out at 300 °C and the NiZ-350 catalyst due to the higher OLP production, an intermediate temperature at which all catalysts and the support exhibited similar OLP and hydrocarbon yields, ranging from 74 to 82%, as shown in Figure 8.

**Figure 8:**
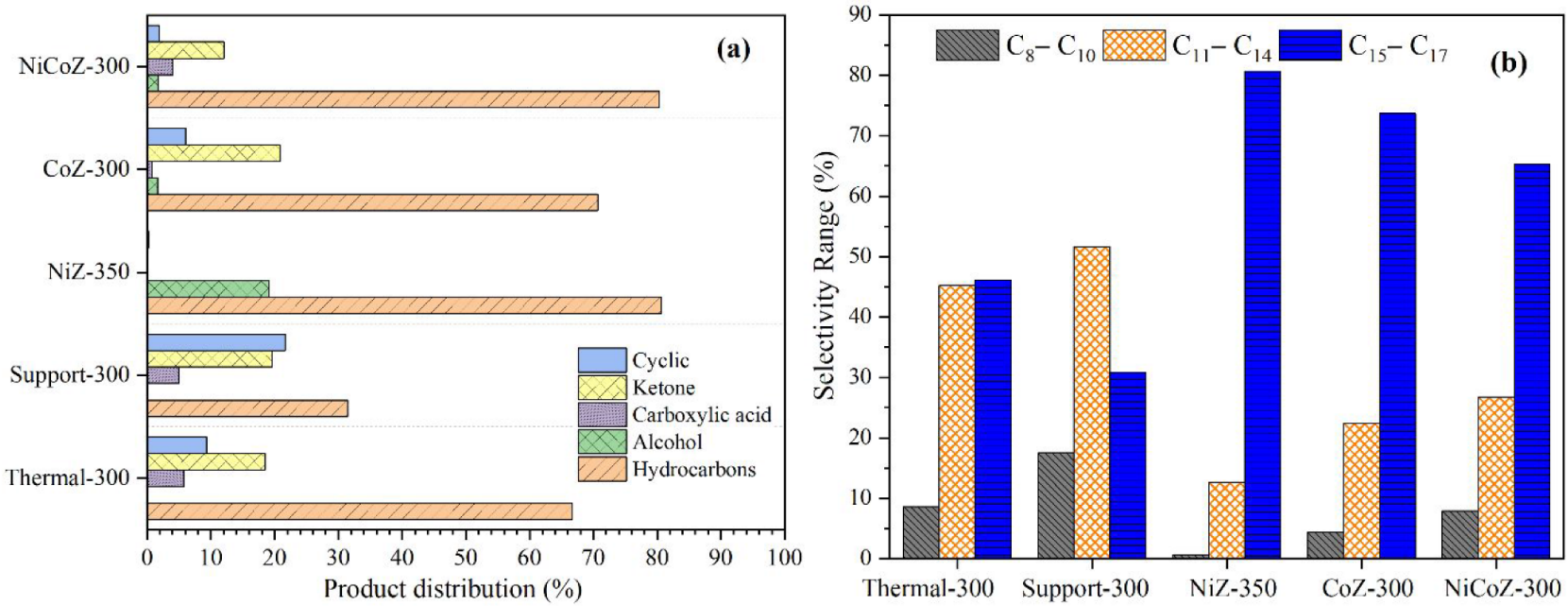
Catalytic activity and selectivity in PFAD deoxygenation reactions: (a) product distribution and (b) hydrocarbon selectivity toward n-(C_8_–C_10_), n-(C_11_–C_14_), and n-(C_15_–C_17_).

As illustrated in Figure 8a, this temperature was chosen to minimize thermal effects and highlight catalytic behavior under equivalent conversion levels. The selectivity data presented in Figure 8b indicates that only the thermal reaction achieved high selectivity toward diesel-range hydrocarbons (C_11_–C_17_), reaching 91% with a nearly equal distribution between the C_11_–C_14_ and C_15_–C_17_ fractions. In contrast, the support exhibited a higher proportion of C_11_–C_14_ and C_8_–C_10_ hydrocarbons, confirming the occurrence of partial cracking of carbon chains during the reaction.^16^

Among the reduced catalysts, NiZ and CoZ exhibited the highest selectivity toward hydrocarbons in the C_15_–C_17_ range (83 and 75%, respectively), indicating their strong promotion of deoxygenation reactions. Previous studies have attributed the catalytic activity of metallic cobalt to its high affinity for oxygen atoms in free fatty acids, which also facilitates CO_2_ adsorption via the decarboxylation pathway due to the interaction between cobalt active sites and oxygenated species.^52,53^ This behavior is consistent with the results reported by Kaewmeesri et al.^54^ and Rashidi et al.,^16^ who employed supports not derived from residual raw materials and likewise demonstrated the effectiveness of Ni- and Co-based catalysts in promoting deoxygenation reactions, reinforcing that even under more sustainable support conditions, these metals remain highly active. In contrast, the bimetallic NiCoZ catalyst showed a moderate decrease in the C_15_–C_17_ fraction, accompanied by an increase in the C_11_–C_14_ and C_8_–C_10_ ranges. This suggests that the simultaneous presence of Ni and Co in the same catalyst may induce partial C–C bond cleavage, leading to changes in the product distribution.

Overall, the results of this study emphasize the decisive role of the metal oxidation state in determining the selectivity of green diesel production from PFAD. Compared with the findings reported by De Oliveira et al.,^5^ the overall conversion to liquid hydrocarbons was similar, but significant differences in product selectivity were observed. While Ni- and Co-supported catalysts in their oxide forms favored the C_15_–C_17_ and C_11_–C_14_ fractions, respectively, their reduced counterparts showed a greater tendency toward the diesel-range hydrocarbons. These observations demonstrate that the oxidation state of the metal on the support directly affects the product distribution and must be carefully considered in the design of more efficient catalysts to improve the quality of green diesel.

### 3.7 Thermodynamic Analysis for Free Fatty Acid Deoxygenation

The conversion pathways of triglycerides and FFAs into hydrocarbons under hydrogen-free conditions using metal-supported catalysts have been addressed in the literature.^5,10,55^ As shown in Table 2, PFAD consists predominantly of FFAs, especially C_16_ and C_18_ species such as palmitic and oleic acids. These compounds can be transformed into hydrocarbons suitable for biofuel applications through decarbonylation and/or catalytic decarboxylation reactions. The proposed reaction pathways and the associated catalytic selectivity during the DO process are illustrated in Figure 9.

**Figure 9:**
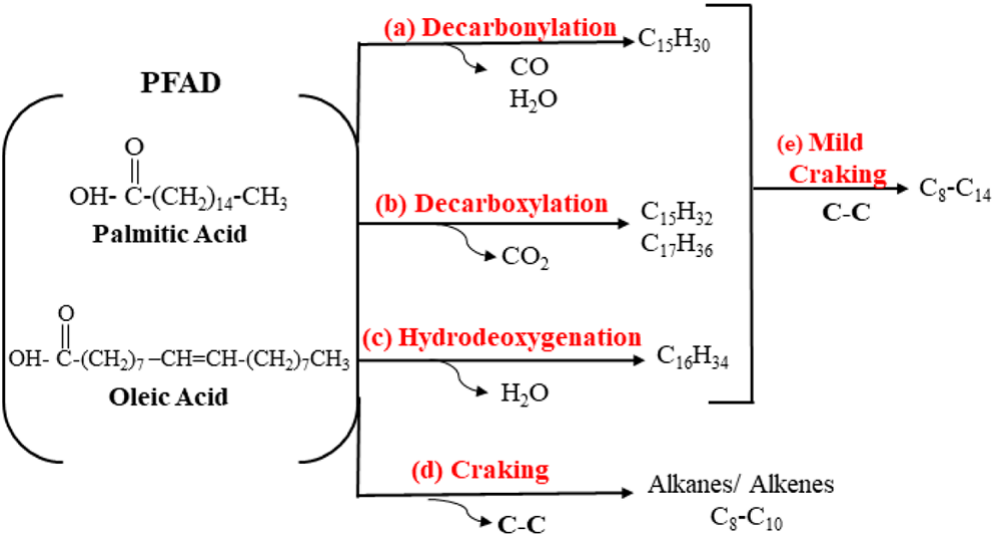
Proposed reaction pathway for PFAD deoxygenation.

Palmitic acid undergoes both decarbonylation (reaction a) and decarboxylation (reaction b), yielding unsaturated pentadecenes (C_15_H_30_) as well as saturated *n*-pentadecane (C_15_H_32_) and *n*-heptadecane (C_17_H_36_), consistent with the mixed C_15_–C_17_ distribution observed. For C_18_ fatty acids, additional pathways contribute: the reverse water–gas shift generates hydrogen that favors alkene hydrogenation, while hydrodeoxygenation produces minor amounts of hexadecanes (reaction c). Concurrently, cracking (reaction d) and C–C scission account for the formation of shorter hydrocarbons and gaseous products.

Catalyst selectivity plays a critical role in determining the product distribution. An effective DO catalyst must remove oxygen efficiently while minimizing carbon loss through C–C bond cleavage, thereby favoring the formation of linear hydrocarbons in the diesel range.^11^ The results of this study indicate that CoZ and NiCoZ exhibited enhanced selectivity toward the decarboxylation pathway, resulting in higher yields of n-C_15_ and n-C_17_. On the other hand, the thermal route and the zeolitic support showed increased activity toward cracking reactions, producing a larger proportion of alkanes, short-chain alkenes, and gaseous compounds.

## 4 Conclusion

The catalytic transformation of PFAD under hydrogen-free and solvent-free conditions demonstrated a cleaner and more sustainable route for producing diesel-range hydrocarbons. The valorization of this agro-industrial residue represents a promising alternative for partially replacing petroleum-derived diesel and can reduce CO_2_ emissions.

Thermogravimetric analysis indicated that PFAD decomposition predominantly occurred between 200 and 320 °C, with the highest mass loss rates observed between 270 and 290 °C, and minimal residual content. The kinetic parameters estimated using the Coats–Redfern method showed activation energies ranging from 91.8 to 119.7 kJ mol^–1^, with lower values observed for the support and NiCoZ. The pre-exponential factor ranged from 3.69 × 10^5^ to 2.37 × 10^8^ s^–1^, suggesting surface-controlled reaction mechanisms.

The catalysts NiZ, CoZ, and NiCoZ showed effective deoxygenation activity, achieving hydrocarbon yields between 77 and 83%, with selectivity for the C_15_–C_17_ diesel fraction ranging from 66 to 80%. The oxidation state of the metal affected the product distribution and, consequently, the quality of the resulting biofuel. This study contributes to the advancement of renewable fuel technologies based on waste-derived feedstocks and low-severity catalytic processes.

## Supplementary Material



## Data Availability

Data available on request from the authors.

## Notes

Gas chromatographic analysis of PFAD composition (Figure S1) (PDF)
