# Supplementary material for: Sustainable Valorization of Palm Fatty Acid Distillate into Green Diesel Using Ni–Co Catalysts Supported on Zeolite from Kaolin Waste under Solvent- and Hydrogen-Free Conditions
Source: ACS Omega. 2025 Nov 8;10(45):54967–77. doi: 10.1021/acsomega.5c08463 (PMC12631470; doi:10.1021/acsomega.5c08463)
Supplement: Supplementary file 1 [file ao5c08463_si_001.pdf]

Supplementary Material for: Sustainable valorization of palm fatty acid distillate into green diesel using Ni-Co catalysts supported on zeolite from kaolin waste under solvent- and hydrogen-free conditions

Brenda Fernanda Honorato de Oliveira[1], Bruno Marques Viegas[2], Mauricio Velasquez[3], Emanuel Negrão Macêdo[1,*]

[1] Engineering of Natural Resources of the Amazon, Federal University of Pará, Belém, PA, 66075-110, Brazil

[2] Graduate Program in Biotechnology, Federal University of Pará, Belém, PA, 66075-110, Brazil

[3] Estado Sólido y Catálisis Ambiental (ESCA), Departamento de Química, Facultad de Ciencias, Universidad Nacional de Colombia, Carrera 30 No. 45-03, Bogotá, Colombia

E-mail addresses

brendafho@ufpa.br (Brenda Fernanda Honorato de Oliveira), viegasmbruno@gmail.com (Bruno Marques Viegas), lmvelasquezma@unal.edu.co (Mauricio Velasquez), enegrao@ufpa.br (Emanuel Negrão Macêdo)

* Corresponding author.

Mailing address: Engineering of Natural Resources of the Amazon, Federal University of Pará, Belém, PA, 66075-110, Brazil

E-mail address: enegrao@ufpa.br (Emanuel Negrão Macêdo)

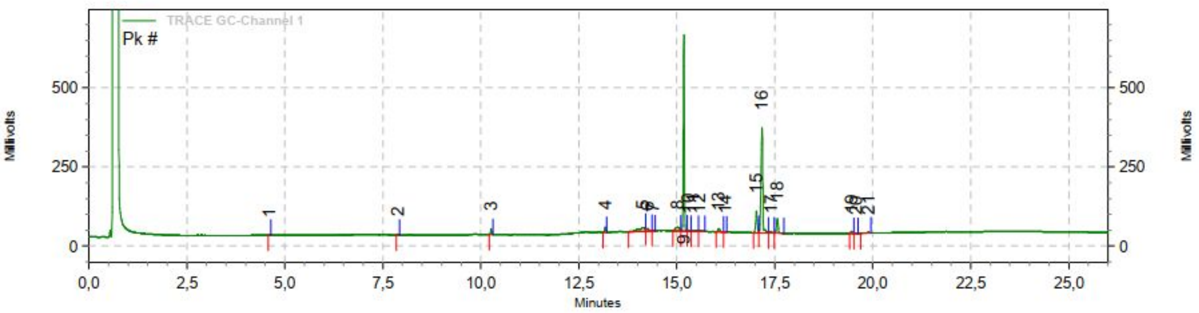

Figure S1 - Gas chromatographic analysis of PFAD composition.
